# Cross-Jurisdictional Sharing for Emergency Management-Related Public Health: Exploring the Experiences of Tribes and Counties in California

**DOI:** 10.3389/fpubh.2017.00254

**Published:** 2017-09-21

**Authors:** Maureen A. Wimsatt

**Affiliations:** ^1^California Tribal Epidemiology Center, California Rural Indian Health Board, Inc., Sacramento, CA, United States

**Keywords:** cross-jurisdictional sharing, public health, emergencies, tribe, county, mixed methods

## Abstract

Each American Indian tribe is unique in several ways, including in its relationships with local governments and risk for emergencies. Cross-jurisdictional sharing (CJS) arrangements are encouraged between tribes and counties for emergency management-related population health, but researchers have not yet explored CJS experiences of tribes and counties for emergency management. This investigation used collaboration theory and a CJS spectrum framework to assess the scope and prevalence of tribe–county CJS arrangements for emergency management in California as well as preconditions to CJS. Mixed-methods survey results indicate that tribes and counties have varied CJS arrangements, but many are informal or customary. Preconditions to CJS include tribe–county agreement about having CJS, views of the CJS relationship, barriers to CJS, and jurisdictional strengths and weaknesses in developing CJS arrangements. Areas for public health intervention include funding programs that build tribal capacity in emergency management, reduce cross-jurisdictional disagreement, and promote ongoing tribe–county relationships as a precursor to formal CJS arrangements. Study strengths, limitations, and future directions are also discussed.

## Introduction

Emergency management is the preparedness, mitigation, response, and recovery efforts of individuals, organizations, communities, and local governments for population health and well-being (1). Seminal perspectives about disasters classified emergencies as acts of God or nature [e.g., Ref. (2)], while social researchers argue that emergencies would be non-existent if not for the intersection between nature and society *via* man-made infrastructure, cultural protections, and societal forces limiting the access of vulnerable populations to resources before, during, or after emergencies (3–6). That is, emergencies would not exist without a society to experience them. Despite the inherent tie between emergencies and societal well-being, early research on emergency management described it as a function of law enforcement instead of a function of governments, public administration, and public health (7).

More recently, researchers have explored emergency management as a measure of public health. For example, policy researchers have described public health preparedness as a broader community, governmental, and societal process and encouraged public health officials to engage in preparedness with outside entities, including local governments (8, 9). Other researchers have examined the associations between emergencies, social contexts, and behavioral outcomes (10). As reviewed by Ejeta and colleagues, scholars have applied the health belief, extended parallel process, and social cognitive models to studies about emergency health preparedness (11), while O’Sullivan and colleagues use complexity theory to suggest that coping with uncertainty, providing surge capacity, being a response organization, and communicating with the public are cross-sectoral, structural emergency management challenges that impact the dynamic system(s) in which emergencies occur (12).

An emerging area of public health research is the study of cross-jurisdictional sharing (CJS) for emergency management. Health and social scientists promote CJS as one collaborative mechanism for sharing resources to address the population health of two or more jurisdictions, particularly in fiscally limited areas of the country and world (13). CJS is important because emergencies do not always occur within one geographic setting or sector, and CJS-guided emergency management across multiple boundaries can increase the effectiveness of preparedness, mitigation, response, and recovery efforts (14, 15). Collaboration theory provides a lens for exploring CJS for emergency management because it emphasizes that there are preconditions which make CJS possible, processes by which CJS occurs, and outcomes of CJS (16). Collaboration theory has guided research about strategic alliances and the need for independent entities to share resources to achieve progress toward mutual goals (17) and can be used as a framework for understanding CJS models like the one proposed by the Center for Sharing Public Health Services (CSPHS), which describes CJS as a deliberate exercise whereby two or more jurisdictions explore, prepare and plan, and implement and improve CJS to deliver essential population health via a spectrum of CJS arrangements. CSPHS suggests that CJS occurs through one or all of the following types of arrangements: informal or customary (sometimes known as as-needed assistance), service-related, shared functions with joint oversight, and/or regionalization (18).

Literature reviews using PubMed, National Institutes of Health Library, and books and journals specific to public health, public administration, community interventions, and emergency management topics (search terms: tribal, governments, American Indian/Alaska Native, emergency management, emergency preparedness, cross-jurisdictional sharing) appear to yield no studies about CJS for emergency management population health between tribal and non-tribal governments. This gap in the empirical literature is problematic because American Indians and Alaska Natives (AIAN) experience disproportionately poorer health outcomes than non-AIAN, all before taking into account the impact of natural and non-natural emergencies on tribal populations (19, 20). The forced relocation of AIAN to rural areas of the United States, far from major hospitals and other emergency resources, contributes to tribes’ vulnerability to emergencies. Social scholars also argue that societal forces place vulnerable populations like tribes at greater risk for harm by emergencies by preventing access to needed resources before, during, and after emergencies (3, 6). For example, in the United States, tribal governments are classified as sovereign nations with the right to engage in federal-to-federal decision-making with the United States government (21). Yet, tribes are not guaranteed access to disaster funds awarded to counties by the federal government and could not directly declare emergencies to the President of the United States until 2013 [i.e., Robert T. Stafford Disaster Relief and Emergency Assistance Act (22)].

Tribal governments may benefit from establishing CJS arrangements with counties to pool resources or formalize assurances that counties will share federal emergency funding, but only a tribe as a self-determined, sovereign governing body can choose to enter into a CJS arrangement with a county (21, 23). Currently, there are tribal governments engaging in CJS to exercise sovereignty regarding the health of their people, including tribes using public health authority, an extension of tribal sovereignty, to meet specific CJS accreditation benchmarks associated with population health (24–28). Some tribes could be hesitant to participate in CJS with county governments due to the historical mistrust between tribal and non-tribal societies, and others may avoid CJS due to limited infrastructure and capacity. However, little is known about the scope, prevalence, and existing conditions for tribe–county CJS for emergency management.

The purpose of this study is to explore the scope and prevalence of tribe–county CJS for emergency management in California, a state with large AIAN and non-AIAN populations susceptible to natural and non-natural emergencies each year (29). This investigation also explores associations between scope and prevalence of CJS and preconditions for CJS, including the size of tribal and county jurisdictions and whether tribes and counties are working toward accreditation in emergency management. Due to historical mistrust between tribal and non-tribal societies, associations are also explored between the scope and prevalence of CJS and whether tribes and counties agree about having CJS arrangements. Qualitative data from a second wave of the study provide insight into three additional preconditions to CJS: Views of the CJS relationship, barriers to tribe–county CJS for emergency management, and jurisdictional strengths and weaknesses in developing CJS arrangements for emergency management.

## Materials and Methods

### Initial CJS Survey

#### Recruitment

Research staff sent a project recruitment letter to the elected Tribal Chairman/woman of 111 federally recognized tribes in California, including the 109 tribes with federal recognition status in 2015 and 2 additional federally recognized tribes from neighboring states with tribal lands in California (30). Follow-up telephone calls and emails were conducted by research staff to finalize a list of participating tribes. A recruitment letter was sent to the Clerk of the Board of Supervisors of any county corresponding to a participating tribe, and research staff undertook methods similar to those for tribes to increase county participation. Tribes and counties were not told which respective county or tribal jurisdiction(s) participated in the study.

#### Participants

Initial CJS survey participants were tribal and corresponding county representatives who received recruitment letters or who were designated to participate. Participants were from 83 of 111 tribal jurisdictions (participation rate = 74.7%) and all 29 counties corresponding to the 83 tribes (participation rate = 100.0%). The 83 participating tribes differed from the 28 non-participating tribes in geographic location [*F*(1,110) = 4.45, *p* < 0.05], with more participants being from northern and central California than southern California (31). Geographic region was not statistically associated with scope and prevalence of CJS arrangements; therefore, the sample of 83 tribal jurisdictions was considered representative.

#### Procedure

After providing informed consent, tribal and county participants completed an Institutional Review Board (IRB)-approved, mixed-methods survey adapted from CSPHS “Existing CJS Arrangement” instrument (32). Questions were tested and refined with an advisory group of tribal and county emergency managers (32, 33), and permission was obtained from CSPHS before using the adapted survey. As is appropriate in studies with AIAN, research staff respected local protocols for data collection, including asking tribal representatives whether they preferred data collection via web-based survey, paper survey, telephone discussion, or in-person discussion (33–35). All tribal representatives chose to complete the survey via telephone or during in-person meetings, with research staff writing down participant responses and reviewing answers with participants to guarantee accuracy. County representatives opted to complete the survey *via* secure internet link. Participants received a small gift after the initial CJS survey and were invited to discuss findings at roundtable meetings after the project ended (36).

#### Measures

Data about participant demographics, jurisdiction-related information, scope and prevalence of CJS arrangements, tribe–county agreement about having no or any CJS, and accreditation-related CJS arrangements were obtained using the initial CJS survey.

##### Demographic Information

Participant role in jurisdiction was an open-ended item about job title/department. Research staff categorized tribal and county responses by department type.

##### Jurisdiction-Related Information

Jurisdiction population and geographic sizes were self-reported by tribal and county representatives. For data analysis, research staff used a California tribal lands directory to derive the total number of tribes in a county (37). Research staff then derived two proportional items: Proportion of total number of tribes per county to county population size and proportion of total tribes per county to county geographic size.

##### Scope and Prevalence of CJS Arrangements for Emergency Management

**Figure 1:**
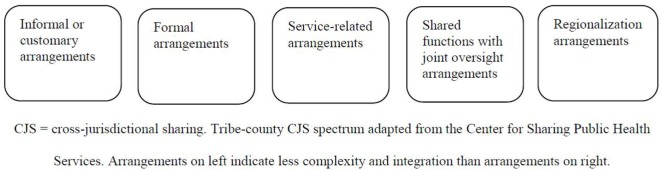
Tribe–county CJS spectrum. CJS, cross-jurisdictional sharing. Tribe–county CJS spectrum adapted from the Center for Sharing Public Health Services. Arrangements on left indicate less complexity and integration than arrangements on right.

In adapting the CSPHS spectrum of CJS arrangements framework (38), initial CJS survey responses were coded into non-mutually exclusive dichotomous variables (0 = *no*, 1 = *yes*) to represent jurisdictions that have: (1) Formal CJS arrangements (1 item; formalized in a written document); (2) informal or customary CJS arrangements (1 item; “handshake agreement,” *not* formalized in a written document); (3) service-related CJS arrangements (1 item; formalized in a contract for as-needed work/consultation); (4) shared functions with joint oversight CJS arrangements (1 item; successful in establishing people from both jurisdictions who make decisions before, during, after emergencies); and/or (5) regionalization arrangements (1 item and response to open-ended comments at end of survey; successful in establishing a group of people from both jurisdictions who make decisions before, during, after emergencies *and* comments that jurisdictions have merged into one department). This coding adaptation is inclusive of CJS arrangements in tribal settings, which can be formal agreements for partnership (e.g., Tribal Resolutions) without specific emergency management tasks and/or informal or customary arrangements unrelated to specific emergency events (see Figure 1).

Research staff reconciled all coding with comments made by participants at the end of the survey. If discrepancies arose between coding and comments, 4-person agreement was reached about how to resolve the inconsistency. For example, if a representative reported no formal CJS arrangements but later stated that the jurisdiction has a tribe–county Memorandum of Understanding for emergency management, the jurisdiction was coded as having formal CJS arrangements. Finally, a sum CJS arrangement score 0–5 was calculated for each jurisdiction.

##### Tribe–County Agreement about CJS Arrangements

Tribe–county agreement about having no or any CJS arrangements for emergency management was cross-tabulated by research project staff. There were four total possible combinations of agreement and disagreement. Agreement was coded when both jurisdictions reported having no (0) or any (1–5) CJS arrangements. Disagreement was coded when one jurisdiction reported having no (0) CJS arrangements, and the other jurisdiction reported having any (1–5) CJS arrangements.

##### Accreditation-Related CJS Arrangements

Jurisdictions had accreditation-related CJS for emergency management when participant reported one reason for establishing the CJS arrangement was to meet national accreditation standards (1 item).

### Follow-up CJS Survey

#### Recruitment, Participants, and Procedure

Research staff narrowed the original sample to 24 representative tribes and 13 corresponding counties (i.e., 6 tribe–county pairs from each CJS agreement and disagreement category in the initial CJS survey). The IRB-approved CJS follow-up survey questions were generated by the advisory group to broadly assess CJS preconditions using open-ended questions (33). After taking the initial CJS survey, tribal and county representatives were recontacted, provided additional informed consent, and answered questions over the telephone. Research staff wrote down participant responses and reviewed written information with participants for accuracy. County representatives were prompted to answer questions for each tribe in their jurisdiction. Thus, 24 tribe and county responses to each item were possible.

#### Measures

Open-ended items on the follow-up CJS survey were about v*iews of tribe–county CJS relationship for emergency management, historical or cultural barriers to the CJS relationship, and strengths and weaknesses of the jurisdiction in developing tribe–county CJS arrangements*. Research staff developed an emergent qualitative coding scheme based on respondents’ answers to follow-up CJS survey items. Emergent themes were added to a preliminary code list using NVivo for Windows Version 11 (39). Independent raters applied the codes, discussed coding discrepancies, and developed new sub-codes if necessary. Raters continued this iterative process until 2-coder agreement was reached for all qualitative data and a final master code list could be applied. For views of the CJS relationship and strengths and weaknesses of the jurisdiction in developing CJS arrangements, codes were applied once. For historical or cultural barriers to the CJS relationship, several participants reported two or more types of barriers. These jurisdictions were coded as having “multiple barriers,” with sub-codes applied for specific themes.

## Results

### Initial CJS Survey

Descriptive statistics were used to assess demographic information, jurisdiction-related information, scope and prevalence of CJS arrangements for emergency management, tribe–county agreement about having no or any CJS arrangements, and accreditation-related CJS arrangements. Correlational and inferential statistics were used to test associations between jurisdiction-related information and scope and prevalence of CJS arrangements; tribe–county agreement about having no or any CJS arrangements and scope and prevalence of CJS arrangements; and accreditation-related CJS arrangements and scope and prevalence of CJS arrangements.

#### Demographic Information

Frequency scores indicated that tribal participants were elected leaders or other tribal administration staff [43.4%; 95% confidence interval (CI) = 32.7, 54.1], tribal emergency managers/staff (37.3%; 95% CI = 26.9, 47.7), tribal environmental managers/staff (16.9%; 95% CI = 8.8, 25.0), or tribal health clinic managers/staff (2.4%; 95% CI = −0.9, 5.7). County participants were Office of Emergency Services staff (86.2%; 95% CI = 73.7, 98.8), health department staff (10.3%; 95% CI = −0.8, 21.4), or administrators (3.4%; 95% CI = −3.2, 10.0%).

#### Jurisdiction-Related Information

Tribes reported serving between 0 (i.e., resident-less) and 84,000 people [M (SD) = 1,650.82 (9,580.64)], with a geographic size ranging from 0 (i.e., currently landless) to 547 square miles [M (SD) = 16.77 (64.88)]. Counties reported serving between 9,500 and 3.2 million people [M (SD) = 468,191.10 (797,677.37)], with a geographic size ranging from 612 to 22,000 square miles [M (SD) = 3,794.24 (4,055.48)]. Total number of tribes per county ranged from 1 to 18 [M (SD) = 7.08 (5.06)]. Proportional variables ranged from 0.000001 to 0.0004 for total number of tribes to county population size [M (SD) = 0.0001 (0.0001)] and 0.0002–0.005 for total number of tribes to county geographic size [M (SD) = 0.003 (0.002)].

#### Scope and Prevalence of CJS Arrangements for Emergency Management

The most frequently reported CJS arrangement for both tribes and counties was informal or customary CJS arrangements, while approximately 15% of tribes and 28% of counties reported having formal CJS arrangements (e.g., Memorandum of Understanding) for emergency management. See Table 1. Overall, tribes ranged between having 0 and 3 CJS arrangements [M (SD) = 0.87 (0.91)], and counties ranged between having 0 and 4 CJS arrangements [M (SD) = 1.48 (1.02)]. A total of 55.4% of tribes (95% CI = 44.7, 66.1) and 75.9% of counties (95% CI = 60.3, 91.5) reported having at least 1 type of CJS arrangement.

**Table 1:** Frequencies and intercorrelations between CJS arrangement variables.

Arrangement type	Tribes (*n* = 83)						Counties (*n* = 29)					
	% (95% CI)	1	2	3	4	5	% (95% CI)	1	2	3	4	5
1. Formal	14.5 (6.9, 22.1)	–	−0.34**	0.29**	0.47***	0.38***	27.6 (11.3, 43.9)	–	−0.58**	0.69***	−0.09	0.35
2. Informal or customary	41.0 (30.4, 51.6)		–	−0.03	0.19	−0.13	55.2 (37.1−73.3)		–	−0.32	0.40*	−0.27
3. Service-related	3.6 (−0.4, 7.6)			–	0.04	−0.03	17.2 (3.5, 30.9)			–	0.07	0.17
4. Shared functions with joint oversight	25.3 (16.0, 34.7)				–	0.27*^a^	41.4 (23.5, 59.3)				–	0.17
5. Regionalization	2.4 (−0.9, 5.7)					–	6.9 (−2.3,16.1)					–

***p < 0.001, **p < 0.01, *p < 0.05.

*CJS, cross-jurisdictional sharing; CI, confidence interval*.

*^a^Small cell size, interpret with caution*.

#### Tribe–County Agreement about CJS Arrangements

Approximately 55% of tribe–county pairs were in agreement about having no or any CJS arrangements, (see Table 2).

**Table 2:** Tribe–county CJS agreement (*n* = 83).

Agreement category	% (95% CI)
Agree: both tribe and county report no CJS arrangements	13.3 (6.0, 20.1)
Agree: both tribe and county report any CJS arrangements	42.2 (31.6, 52.8)
Disagree: county reports CJS arrangements but tribe does not	31.3 (21.3, 41.3)
Disagree: tribe reports CJS arrangements but county does not	13.3 (6.0, 20.1)

*CJS, cross-jurisdictional sharing; CI, confidence interval*.

#### Accreditation-Related CJS Arrangements

A total of 3.6% of tribes (95% CI = −0.4, 7.6) and 6.9% of counties (95% CI = −2.4, 16.0) reported that CJS arrangements were to meet national accreditation standards.

#### Associations between Jurisdiction-Related Information and Scope and Prevalence of CJS Arrangements

There were significant positive associations between three jurisdiction-related variables and specific types of CJS arrangement. For tribes, there were significant positive associations between population size and having formal CJS arrangements (*r* = 0.24, *p* < 0.05); population size and having shared functions with joint oversight CJS arrangements (*r* = 0.25, *p* < 0.05); and geographic size and having shared functions with joint oversight CJS arrangements (*r* = 0.24, *p* < 0.05). For counties, there was a significant positive association between total number of tribes per county and having informal or customary CJS arrangements (*r* = 0.43, *p* < 0.05).

Multiple linear regression analyses revealed one significant negative association between proportion of tribes per county to county population size and tribe-reported sum of CJS arrangements (*t* = −2.22, *p* < 0.05): a greater total number of tribes to county population size were associated with fewer total CJS arrangements reported by tribes. There were no significant associations between proportional variables and county-reported prevalence of CJS arrangements.

#### Associations between Tribe–County Agreement about CJS and Scope and Prevalence of CJS Arrangements

Chi-square and simple linear regression analyses determined that tribe–county agreement about having no or any CJS arrangements was positively associated with tribe-reported formal CJS arrangements [χ^2^(1) = 4.42, *p* < 0.05], informal or customary CJS arrangements [χ^2^(1) = 7.64, *p* < 0.01], and shared functions with joint oversight CJS arrangements [χ^2^(1) = 7.42, *p* < 0.01]. Tribe–county agreement was also positively associated with tribe-reported prevalence of CJS arrangements (β = 0.79, *t* = 4.32, *p* < 0.001). Tribe–county agreement about having no or any CJS arrangements was not significantly associated with county-reported scope or preference CJS arrangements.

#### Associations between Accreditation-Related CJS Arrangements and Scope and Prevalence of CJS Arrangements

Given the small number of tribes and counties that reported having CJS arrangements to meet national accreditation standards, it was not possible to statistically test associations between accreditation-related CJS arrangements and scope and prevalence of CJS arrangements.

### Follow-up CJS Survey

Qualitative analyses were conducted to determine emergent themes in response to three follow-up CJS survey items. Descriptive statistics were used to tabulate how frequently themes were reported for each item.

#### Views of the Tribe–County CJS Relationship

When asked to describe views of the tribe–county CJS relationship for emergency management, representatives from 24 tribes and 13 corresponding counties provided emergent themes of *positive, negative, neutral*, and *non-existent*. Positive views were limited to comments depicting the tribe and county as having a strong or collaborative relationship. Neutral views were comments without a discernable positive or negative sentiment. Non-existent views were comments about the tribe and county having no relationship. Negative views were comments expressing displeasure or distrust about the other jurisdiction.

A total of eight tribal representatives reported having a neutral CJS relationship with the county, while seven had a negative relationship, six had a positive relationship, and three had a non-existent relationship. County representatives reported 11 neutral, 7 positive, and 6 non-existent CJS relationships with tribes. No county representatives reported having a negative CJS relationship with a tribe. Example views include:

The overall relationship with the tribe is great and open. There is a current Memorandum of Understanding in place with the tribe and [nearby] hospitals. (County, positive)

Overall the relationship is neutral. There is an agreement for emergency services on tribal lands [called the] Economic Development Enterprise, a.k.a. Gaming Compact, but if there is a level of conversation at the county level about emergency services, the tribe is not at the table or made aware of the meetings. The tribal council hasn’t made it a priority to ask the county about these meetings. (Tribe, neutral)

There is no ongoing relationship between the tribe and county. (Tribe, non-existent)

Any time the [surrounding] tribes start casinos, the county tries to benefit. The tribe is skeptical about outreach [to attend emergency management and preparedness meetings] because the tribe feels the county is trying to meet a requirement. If there were a natural disaster, the tribe would feel uncomfortable and would be skeptical about whether or not state or county services would be provided to the tribe. (Tribe, negative)

Because only tribes reported having a negative view of the CJS relationship, tribal and county views were sometimes disparate:

The relationship is non-existent. The county as a whole hasn’t really heard from the tribe since the flood [omitted] when the tribe lost property. (County, non-existent)

The tribe had experienced high waters and nobody from the county came to check on our well-being or alert tribal members. (Tribe, negative)

#### Historical or Cultural Barriers to the CJS Relationship

Emergent themes regarding historical or cultural barriers to the CJS relationship for emergency management were *legal/jurisdictional restrictions, distrust, limited knowledge about tribal systems, multiple, other*, and *no/unknown*. Legal/jurisdictional barriers were present when a respondent noted laws (e.g., Public Law 83-280 known as “PL280”) and/or governmental limitations to the tribe–county CJS relationship. Distrust was comments referencing current or historical mistrust between the tribe and county, including non-tribal governments participating in the massacre and displacement of tribal peoples. Limited knowledge of tribal systems was comments about limited or lacking knowledge about tribal sovereignty, tribal government and infrastructure (e.g., funding and tribal jobs/roles), or cultural protocols. Multiple barriers were present for jurisdictions with two or more of the aforementioned barriers, and other barriers were beyond an emergent theme. No/unknown barriers were coded for tribal and county jurisdictions with no known restrictions. Tribes most frequently reported multiple barriers to the CJS relationship for emergency management, while counties most frequently reported no/unknown barriers. See Tables 3 and 4 for frequency scores and examples by theme.

**Table 3:** Tribe- and county-reported barriers to CJS by theme.

Theme	Tribes (*n* = 24)	Counties (*n* = 24)^a^
Legal/jurisdictional restrictions	4	2
Distrust	0	5
Limited knowledge of tribal systems	3	2
Multiple barriers^b^	8	1
Other^c^	3	3
No/unknown	6	11

*CJS, cross-jurisdictional sharing*.

*^a^n = 24 represents 13 counties reporting about each of the 24 tribes*.

*^b^Multiple barriers include two or more of legal-jurisdictional, distrust, and/or limited knowledge of tribal systems barriers*.

*^c^Other barriers were lack of funding, geographic isolation, and having one of point of contact within the tribe*.

**Table 4:** Examples of tribe- and county-reported barriers to CJS by theme.

Theme	Example
Legal/jurisdictional restrictions	*The relationship that California and its tribes have in emergency management with Public Law 280 status is a barrier. Tribes wish to interact, but city and county groups do not based on Public Law 280, [and] tribes are left out of emergency management planning. Public Law 280 affects tribal law enforcement greatly on the California side as tribes have no authority on tribal land and have to work jointly with the county even while on tribal lands*. (Tribe) *[Lack of] water rights and the process of water rights*. (Tribe) *Historically, the tribal police department is federally recognized, which means if anyone is arrested on the tribe’s land, they must go to a federal prison. The closest federal prison is in Sacramento. Once [a person is] in the federal prison, federal charges have to be filed, which is rare in Sacramento courts. Because of the [geographic] distance and barriers involving Sacramento, a lot of times the county sheriff will have to go to the tribe and can only do a citizen’s arrest*. (County)
Distrust	*The major historical barrier with the tribe is the major distrust of white people due to the massacres [which took place 1851–56]. The massacres have never been forgotten or forgiven*. (County) *Historically the tribe was displaced by the Army Corp of engineers*. (County)
Limited knowledge of tribal systems	*During the fall fires, work was being done before the acknowledgment that cultural resources were destroyed and damaged by the fires and cleanup. There was no communication or funding for cultural monitors. The county also did not understand the importance of watershed monitoring*. (Tribe) *Culturally, the tribe feels the county and others did not respect their sovereignty until the tribe started gaming […]*. (Tribe) *One cultural barrier is when one person plays different roles as the Tribal Council member and as a tribal employee. You have to know which role the person is in at that moment, which can affect how things are approached regarding emergency management*. (County)
Multiple barriers	*There is a misunderstanding in the county about sovereignty and Public Law 280. The city officials all over the county do not seem to understand Indian reservations and/or the relationship between the tribes and the U.S. government*. (Tribe, legal/jurisdictional restrictions and limited knowledge of tribal systems) *Standardized Emergency Management System (SEMS) affects how the tribe is viewed by the county since SEMS doesn’t recognize the tribe’s operational areas. Tribes are included in SEMS but not tribal lands. In the past twenty years, it has had an effect on tribal emergencies but in the past five years, the state and counties are making efforts to solve this [confusion] with California Office of Emergency Service tribal liaisons. Historically, there is past poor treatment by counties. The tribe usually has to be the one to initiate talks with the county*. (Tribe, legal/jurisdictional restrictions and distrust) *California state law does not recognize the tribes as sovereign. There is a lack of recognition of the tribes when it comes to joint power agreements, like auto aid or mutual aid agreements. This sets the tribe up for failure. There is a bias on both parts, tribe and county. Tribes have a lack of trust for the county. The county does not recognize tribal contributions and capabilities*. (Tribe, legal/jurisdictional restrictions, distrust, and limited knowledge of tribal systems) *There is a deep-rooted ongoing distrust on behalf of the tribe. The county is trying to establish a mutual aid arrangement (MAA) with the tribe. The tribe is concerned with how the MAA will impact other arrangements in place and the tribe’s sovereignty. The tribe won’t discuss changing the language or other options for the MAA. Instead of working with the county, the tribe tends to shut down. I have observed an overly strong knee-jerk guarding reaction from the tribe, but I believe the reaction is warranted due to past historical treatment*. (County, distrust and limited knowledge of tribal systems)
Other	*The concerns come down to funding. Bigger tribes like [omitted] have a Memorandum of Understanding with the county as well as the tribal infrastructure, including tribal fire departments, but the tribes still pay the county for services. Since the smaller tribes either don’t have casinos or don’t have successful ones, the county seems to be less interested because the Memorandum of Understanding will not provide funding for the county. The county has a mentality that tribes should pay a fair share […]*. (Tribe) *I am cautious of only having one point of contact within the tribe*. (County)
No/unknown	*Not aware of anything*. (Tribe) *Unsure. To say anything would be assuming*. (County)

*CJS, cross-jurisdictional sharing*.

#### Strengths and Weaknesses of the Jurisdiction in Developing Tribe–County CJS Arrangements

A total of 14 tribal representatives and 13 county representatives identified strengths of their own jurisdiction in developing CJS arrangements for emergency management. *Relationships* and *jurisdictional preparedness* were tribal strengths, and *relationships* and *resources* were county strengths. Relationship strengths were coded for 11 tribal and 12 county representatives who said that their jurisdiction’s strength was networking, engaging in ongoing CJS communications, or collaborating on tribe–county projects to develop CJS arrangements:

[Our strength is] working with the county on the tribe’s Hazard Mitigation Plan. Emergencies bring people together. (Tribe, relationships)

The current sheriff goes out and meets with [omitted] Tribe each month and does community meetings annually where all tribes in the county are invited. (County, relationships)

A total of three tribal participants said their jurisdiction’s strength was being ready for an emergency (i.e., jurisdictional preparedness), and one county representative said their jurisdiction’s strength was its resources:

The tribe has a good standalone emergency management program. (Tribe, jurisdictional preparedness)

The county has resources that tribes can access. (County, resources)

A total of 10 tribal and 7 county representatives identified weaknesses of their own jurisdiction in developing CJS arrangements. For tribes, *limited or no capacity for emergency management* was a weakness reflected in statements about limited tribal training, funding, or manpower for emergency management work.

There are time restrictions on tribal staff. (Tribe, limited or no capacity)

Sometimes the capacity of the tribe isn’t large enough to support the full structure that may be required [for emergency management]. (Tribe, limited or no capacity)

The tribe needs to convince their elected officials that money needs to be spent in this area. (Tribe, limited or no capacity)

For counties, jurisdictional weaknesses were *laws that restrict the development of CJS arrangements*, having *limited staff* for *emergency management* (“the county is scrambling”), and *lack of information*, including not knowing who to contact within a tribe to initiate CJS, tribal geographic information, or why the CJS relationship had not advanced:

[My weakness is that] I cannot seem to develop relationships with the tribes in the county. If there is contact possible, I would like to have it. (County, lack of information)

One unexpected set of findings emerged during qualitative analyses because some tribal and county jurisdictions reported weaknesses of the other jurisdiction in addition to weaknesses of their own jurisdiction. Tribal representatives said that county representatives exclude tribes from emergency management trainings and events. County representatives reported that tribes have frequent staff turnover and do not have designated tribal emergency managers who can help develop tribe–county CJS arrangements (“tribal staff wear many hats”). One county representative cited lacking formal arrangements as a weakness of tribes. Examples include:

The tribe is never sent an official invitation to the emergency management development meetings hosted by the county, even though the tribe would like to attend. (Tribe, discussing county weakness)

It’s a challenge that most tribes do not have an established emergency manager, often because of [not having] funding. If there is an emergency manager within the tribe, sometimes it is someone who has multiple responsibilities and won’t have time to engage in the advanced planning for emergency management. (County, discussing tribe weakness)

The lack of formal written agreements [is a weakness] because relying on handshakes [with tribes] can be iffy. (County, discussing tribe weakness).

## Discussion

This study provides novel information about tribe–county CJS experiences for emergency management and population health. This is the first empirical investigation to use collaboration theory and the adapted CSPHS CJS spectrum to explore the scope, prevalence, and preconditions of CJS between tribes and counties for emergency management. Results indicate that CJS arrangements vary in scope, with all five types of arrangements reported by California jurisdictions with existing tribe–county CJS for emergency management. Less integrated CJS arrangements were predominant in this sample, with both tribes and counties most frequently reporting informal or customary CJS arrangements (18). This finding supports literature about tribal leaders not wanting to establish written arrangements so they can maintain flexibility in cross-sectoral responsibilities and/or allow time for the development of trust between tribal and non-tribal partners (27). It is noteworthy, however, that qualitative data from this study highlight limited tribal capacity and funding for emergency management as preconditions which weaken tribe–county CJS. Additional findings reveal that only tribes with larger population sizes are associated with having formal CJS arrangements. Thus, although CJS is a mechanism by which resource-limited jurisdictions can pool manpower and funding to improve population health (13, 18), results of this study suggest that smaller tribal jurisdictions may seek and benefit from informal or customary CJS arrangements because they do not possess the minimal emergency management assets required to enter into written tribe–county agreements.

Because nearly 20% of the total number of United States federally recognized tribes are located in California, results of this study offer preliminary understanding about the nationwide prevalence of tribe–county CJS for emergency management (30). Findings suggest that more than half of tribes and three-fourths of counties report having at least one CJS arrangement for emergency management. Yet, results also highlight a gap among tribes and counties with no CJS arrangements. Statistical analyses further indicate that tribes report fewer total CJS arrangements when located in counties with a greater total number of tribes to county population size. In these areas, the tribe–county CJS relationship could be diminished because county representatives assume there are enough other people to help tribes before, during, or after emergencies (40). However, due to the unpredictability of emergencies, county representatives do not know whether tribal resources (e.g., shelter, food, and staff) will be the only means by which to protect non-tribal citizens (41). It would benefit tribes and counties to formulate CJS arrangements to protect both populations.

Another important finding is the discordance between tribal and county perspectives about having CJS for emergency management. Nearly half of tribes and counties disagree about working together, with disagreement most frequently occurring when counties report CJS but tribes do not. Different governmental priorities and an inability or limited desire to communicate contribute to disagreement between tribal and non-tribal representatives, but increased meaningful interactions and mutual understanding of respective jurisdictional values can mitigate conflicting perspectives (42). Results of this study also suggest that tribe–county CJS agreement is associated with tribe-reported scope and prevalence of CJS, signifying the importance of concordance as a precondition for tribal CJS experiences. In turn, population health efforts should be aimed at facilitating long-term, tribe–county CJS collaborations that increase mutual awareness, decrease disagreement, and establish successful CJS arrangements (43).

The mixed methodology of this study allows for both systematic and emergent identification of preconditions to CJS. As previously mentioned, jurisdiction-related indicators (i.e., jurisdiction size and relation to total number of tribes in county) and tribe–county CJS agreement are statistically associated with scope and prevalence of CJS arrangements for emergency management. Qualitative data offer nuanced information about three additional preconditions to CJS. Specifically, views of the CJS relationship, barriers to CJS, and jurisdictional strengths and weaknesses in developing CJS arrangements seem to impact jurisdictions’ willingness and ability to participate in tribe–county CJS. Emergent themes reveal varying sentiments about the CJS relationship (i.e., positive, negative, neutral); jurisdiction-specific strengths and weaknesses in developing CJS arrangements (e.g., relationship building and limited capacity); and weaknesses of the other jurisdiction in tribe–county CJS (e.g., tribal staff turnover, preference for informal arrangements, exclusion by counties). Tribes and counties report barriers to CJS that range from systems-level legal issues like Public Law 280, which contributes to tensions between tribal and county law enforcement in at least six states (44, 45), to distrust, limited knowledge of tribal systems by county representatives, and a combination of these barriers. In the future, researchers can utilize emergent themes to further explore direct and indirect relations between preconditions to CJS and scope and prevalence of tribe–county CJS arrangements for emergency management. This will be profitable when examining themes with sub-categorizations that vary by jurisdiction type, including negative views of the CJS relationship which were only reported by tribes and no/unknown barriers to the CJS relationship which were frequently reported by counties but not tribes. Additional mixed-methods research with regional and national samples of tribes and counties will improve understanding about tribe, county, and tribe–county (i.e., actor–partner) models of CJS preconditions and arrangements in the United States.

There is interest in traditional, practice-based accreditation for population health throughout Indian Country, and some tribes report developing CJS arrangements to meet national accreditation standards (27, 28, 46). In this study, however, accreditation-related CJS arrangements were not common or relatable to scope and prevalence of tribe–county CJS. One explanation for this finding is that tribes and counties value CJS for emergency management above and beyond meeting accreditation requirements. Another possibility is that tribes have difficulty aligning emergency management, population health, and accreditation efforts with national benchmarks. In California, for example, tribal governments can be involved in emergency management and also designate health functions to tribal health programs (23). Unfortunately, neither tribal government nor health systems are typically compatible with the organizational structures required to achieve national accreditation (47). In this study, lacking accreditation-related CJS arrangements highlight an opportunity for population health programs to advance tribal capacity for accreditation and tribe–county CJS for emergency management.

Strengths of this study include using culturally responsive research methodology and social theory to examine tribe–county CJS for emergency management. Tribal Epidemiology Center staff with experience navigating tribal protocols used an iterative process for study improvement which involved seeking guidance from advisory group members and tribal leaders during project recruitment, data collection, and dissemination (48). Adapting data collection methods can contribute to higher participation rates by tribes because protocols respect and adhere to sovereign preferences for research within tribal nations (33). By grounding this study in collaboration theory and the CJS framework, results also contribute to the literature about emergency management as a faction of societal health and well-being and expand our knowledge about tribal and county representatives from varied disciplines who participate in emergency management to protect the health of their people (7).

One limitation of the present research is the assumption that CJS is a linear process in which preconditions are antecedents to the scope and prevalence of tribe–county CJS arrangements for emergency management. Instead this process may be interdependent in nature, and future investigations should explore this possibility. The purpose of this study was to examine CJS for emergency management between federally recognized tribes and corresponding counties, but state-recognized tribal governments may have disparate experiences with CJS given their status as non-federal entities (49). Thus, future research should address the second limitation of this study, which is the exclusion of state-recognized tribes. Finally, results and generalizability of this investigation, including qualitative data, would be enhanced by mixed-methods explorations of tribe–county CJS for emergency management across the United States. At the time of this publication, there were 567 federally recognized tribes (50), and it may be that region- and community-specific best practices and experiences, including information unpublished in the formal empirical literature, could vary in places like frontier Alaska Native villages, which represent approximately 40% of tribes nationwide. Future studies about tribe–county CJS for emergency management would provide a clearer picture of the generalizability of this study.

## Conclusion

This study used a public health framework to examine tribe–county CJS experiences for emergency management and preconditions to CJS arrangements. Quantitative and qualitative findings suggest that tribes and counties have varied arrangements for CJS, many of which are informal or customary. Preconditions to tribe–county CJS are jurisdiction-related indicators, tribe–county CJS agreement, views of the tribe–county CJS relationship, barriers to tribe–county CJS, and strengths and weaknesses of jurisdictions in developing CJS arrangements. Areas for public health intervention include programs that build tribal capacity in emergency management and accreditation, reduce cross-jurisdictional disagreement, and promote ongoing relationship building between tribes and counties as a precursor to formal CJS arrangements. Future public health practice and research initiatives about tribe–county CJS for emergency management can help reduce emergency-related health disparities among AIAN people and protect both tribal and county jurisdictions before, during, and after emergencies.

## Ethics Statement

This study was carried out in accordance with the recommendations of the California Rural Indian Health Board, Inc. (CRIHB) Institutional Review Board (IRB) with written informed consent from all subjects. All subjects gave written informed consent in accordance with the Declaration of Helsinki. The protocol was approved by the CRIHB IRB.

## Author Contributions

MW served as the principal investigator of this project and participated in all aspects of research, including developing surveys; facilitating California Rural Indian Health Board, Inc. (CRIHB) IRB approvals; planning and conducting project advisory group meetings; collecting on-site, telephone, and web-based data from tribes and counties; supervising staff in data collection; conducting quantitative and qualitative analyses to test research questions; and writing this manuscript. MW circulated the document for review within CRIHB and received approval of the manuscript from the CRIHB IRB.

## Conflict of Interest Statement

The author declares that the research was conducted in the absence of any commercial or financial relationships that could be construed as a potential conflict of interest.

## References

[1] Federal Emergency Management Agency (FEMA). National Preparedness Goal (2015). Available from: https://www.fema.gov/media-library-data/1443799615171-2aae90be55041740f97e8532fc680d40/National_Preparedness_Goal_2nd_Edition.pdf

[2] Burton I Kates RW The perception of natural hazards in resource management. Nat Resour J (1964) 3(3):412–41.

[3] Cannon T Vulnerability analysis and the explanation of ‘natural’ disasters. In: Varley A, editor. Disasters, Development, and Environment. Wiley & Sons (1994). p. 13–30.

[4] Carr LT Disasters and the sequence-pattern concept of social change. Am J Sociol (1932) 38:207–18. 10.1086/216030

[5] Dombrowsky WR Again and again: is a disaster what we call “disaster”? Some conceptual notes on conceptualizing the objects of disaster sociology. Int J Mass Emerg Disasters (1995) 13:241–54.

[6] Tobin GA Bell HM Whiteford LM Montz BE Vulnerability of displaced persons: relocation park residents in the wake of Hurricane Charley. Int J Mass Emerg Disasters (2006) 24(1):77–109.

[7] Petak WJ Emergency management: a challenge for public administration. Public Adm Rev (1985) 45:3–7. 10.2307/3134992

[8] Lurie N Nelson CD Wasserman J. Public health preparedness: evolution or revolution? Health Aff (2006) 25:935–45. 10.1377/hlthaff.25.4.935 16835172

[9] Nelson C Lurie N Wasserman J Zakowski S Leuschner KJ Conceptualizing and Defining Public Health Emergency Preparedness. Santa Monica, CA: RAND Corporation (2008). 10.2105/AJPH.2007.114496 PMC1854988 17413078

[10] Rung AL Gaston S Oral E Robinson WT Fontham E Harrington DJ Depression, mental distress, and domestic conflict among Louisiana women exposed to the deepwater horizon oil spill in the WaTCH study. Environ Health Perspect (2016) 124:1429–35. 10.1289/EHP167 27164620 PMC5010393

[11] Ejeta LT Ardalan A Paton D. Application of behavioral theories to disaster and emergency health preparedness: a systematic review. PLoS Curr (2015) 7: 1–28. 10.1371/currents.dis.31a8995ced321301466db400f1357829 26203400 PMC4494855

[12] O’Sullivan TL Kuziemsky CE Toal-Sullivan D Corneil W. Unraveling the complexities of disaster management: a framework for critical social infrastructure to promote population health and resilience. Soc Sci Med (2013) 93:238–46. 10.1016/j.socscimed.2012.07.040 22898721 PMC7115777

[13] Shah GH Badana A Robb C Livingood WC Cross-jurisdictional resource sharing in changing public health landscape: contributory factors and theoretical explanations. J Public Health Manage Prac (2016) 22:110–9. 10.1097/PHH.0000000000000368 26808685

[14] Ansell C Boin A Keller A Managing transboundary crisis: identifying the building blocks of an effective response system. J Contingencies Crisis Manage (2010) 18:195–207. 10.1111/j.1468-5973.2010.00620.x

[15] Waugh WL Streib G Collaboration and leadership for effective emergency management. Public Adm Rev (2006) 66:131–40. 10.1111/j.1540-6210.2006.00673.x

[16] Gray B Wood DJ Collaborative alliances: moving from practice to theory. J Appl Behav Sci (1991) 27:3–22. 10.1177/0021886391271001

[17] Gajda R Utilizing collaboration theory to evaluate strategic alliances. Am J Eval (2004) 25:65–77. 10.1016/j.ameval.2003.11.002

[18] Center for Sharing Public Health Services (CSPHS). Spectrum of Cross-Jurisdictional Sharing Arrangements (2017). Available from: http://phsharing.org/2017/03/10/spectrum-of-cross-jurisdictional-sharing-arrangements/

[19] Nelson SE Wilson K. The mental health of Indigenous peoples in Canada: a critical review of research. Soc Sci Med (2017) 176:93–112. 10.1016/j.socscimed.2017.01.021 28135694

[20] Sarche M Spicer P. Poverty and health disparities for American Indian and Alaska Native children: current knowledge and future prospects. Ann N Y Acad Sci (2008) 1136:126–36. 10.1196/annals.1425.017 18579879 PMC2567901

[21] United Nations. United Nations Declaration of the Rights of Indigenous Peoples (2007). Available from: http://www.un.org/esa/socdev/unpfii/documents/DRIPS_en.pdf

[22] Robert T. Stafford Disaster Relief and Emergency Assistance Act. (2013). Available from: https://www.fema.gov/media-library-data/1490360363533-a531e65a3e1e63b8b2cfb7d3da7a785c/Stafford_ActselectHSA2016.pdf

[23] Indian Self-Determination and Educational Assistance Act. (2006). Available from: https://www.bia.gov/sites/bia.gov/files/assets/public/doc/idc-006803.doc

[24] Bertolli J Chao E Landen M Wells E Hayes JM Mahal Z Multistate assessment of public health surveillance relevant to American Indians and Alaska Natives, 2007. J Health Dispar Res Prac (2011) 5(1):99–109.

[25] Bryan R Schaefer RM DeBruyn L Stier D. Public health legal preparedness in Indian Country. Am J Public Health (2009) 99:607–14. 10.2105/AJPH.2008.146522 19150897 PMC2661496

[26] Bullard CH Hogan RD Penn MS Ferris J Cleland J Stier D Improving cross-sectoral and cross-jurisdictional coordination for public health emergency legal preparedness. J Law Med Ethics (2008) 36:57–63. 10.1111/j.1748-720X.2008.00262.x 18315754

[27] Institute for Wisconsin’s Health, Inc. Exploring Service Sharing to Improve Tribal Public Health (2014). Available from: http://phsharing.org/wp-content/uploads/2015/01/TSSBriefFINAL.pdf

[28] Redstar Innovations. Tribal State Relations in Public Health (2014). Available from: http://www.redstarintl.org/wp-content/uploads/PracticeBrief_121014_FINAL.pdf

[29] FEMA. Indian Health Program: Emergency Preparedness Public Private Partnership California (2015). Available from: https://www.fema.gov/pdf/privatesector/ca_ps_tribal.pdf

[30] United States Department of the Interior. Federally Recognized Tribes in California (2015). Available from: http://www.cgcc.ca.gov/documents/Tribal/2015/Federally_Recognized_Tribes_6-15-15.pdf

[31] Indian Health Service. California Health Programs (2017). Available from: https://www.ihs.gov/california/index.cfm/health-programs/health-programs/

[32] CSPHS. Assessment Tool for Public Health: Existing CJS Arrangements Detailed Survey (2016). Available from: http://www.phsharing.org/wp-content/uploads/2014/01/PDFAssessmentOfCJSArrangementsDetailedV1.pdf

[33] Fisher PA Ball TJ. Balancing empiricism and local cultural knowledge in the design of prevention research. J Urban Health (2005) 82:ii44–55. 10.1093/jurban/jti063 15933330 PMC3455902

[34] Weaver HN. Assessing the needs of Native American communities: a Northeastern example. Eval Program Plann (1999) 22:155–61. 10.1016/S0149-7189(99)00010-5 24011410

[35] Nielsen MO Gould LA Non-native scholars doing research in Native American communities: a matter of respect. Soc Sci J (2007) 44:420–33. 10.1016/j.soscij.2007.07.002

[36] Letiecq B Bailey SJ. Evaluating from the outside: conducting cross-cultural evaluation research on an American Indian reservation. Eval Rev (2004) 28:342–57. 10.1177/0193841X04265185 15245624

[37] California Governor’s Office of the Tribal Advisor. Directory of Tribal Governments. West Sacramento, CA: California Governor’s Office of the Tribal Advisor (2015).

[38] CSPHS. A Roadmap to Develop Cross-Jurisdictional Sharing Initiatives (2016). Available from: http://phsharing.org/roadmap/

[39] NVivo for Windows Version 11. Qualitative Data Analysis Software. QSR International Pty Ltd (2015).

[40] Latané B Nida S Ten years of research on group size and helping. Psychol Bull (1981) 89:308–24. 10.1037/0033-2909.89.2.308

[41] California Tribal Epidemiology Center. Toolkit: Cross-Jurisdictional Sharing between Tribes and Counties for Emergency Management. Sacramento, CA: California Rural Indian Health Board Inc. (2017).

[42] TallBear K Understanding the Federal/Tribal Relationship and Barriers to Including Tribes in Environmental Decision-Making. Denver, CO: International Institute for Indigenous Resource Management (2001).

[43] Carroll J Rosson MB Farooq U Xiao L Beyond being aware. Inform Organ (2009) 19:162–85. 10.1016/j.infoandorg.2009.04.004

[44] Wimsatt MA Smith C Wilbur A National Policy Matrix: Cross-Jurisdictional Sharing Arrangements between Tribes and Counties. Sacramento, CA: California Tribal Epidemiology Center, California Rural Indian Health Board, Inc. (2015).

[45] Hernandez-Santana A Barriers to Collaboration between Tribal and County Governments: Planning for Major Disasters and Other Emergencies. Sacramento, CA: California Tribal Epidemiology Center, California Rural Indian Health Board, Inc. (2017).

[46] National Indian Health Board (NIHB). Traditional and Evidence-Based Practices in Public Health. Washington, DC: NIHB (2017).

[47] NIHB. Exploring Tribal Public Health Accreditation: Benefits, Challenges, and Opportunities. Washington, DC: NIHB (2017).

[48] Tribal Epidemiology Centers. (2017). Available from: https://tribalepicenters.org/about/.

[49] National Conference of State Legislatures. Federal and State Recognized Tribes (2017). Available from: http://www.ncsl.org/research/state-tribal-institute/state-recognition-of-american-indian-tribes.aspx

[50] Bureau of Indian Affairs. Indian Entities Recognized and Eligible to Receive Services from the United States Bureau of Indian Affairs (2017). Available from: https://www.gpo.gov/fdsys/pkg/FR-2017-01-17/pdf/2017-00912.pdf

